# Thoughts on Decay and Its Treatment

**Published:** 1860-05

**Authors:** Geo. S Fouke


### THOUGHTS ON DECAY AND ITS TREATMENT.

BY GEO. S. FOUKE.

WE have suggested to the profession the following names for the stages of dental decay, namely, *Epienamel* and *Subenamel* decay; and *Epidentinal* and *Infradentinal* for decays existing on or in the dentinal tissue. We would express a decay reaching to the living membrane of the pulp by the term *Subdentinal*, which would make the nomenclature complete, and render the vague terms "superficial" and "deep-seated," obsolete as dental technicalities.

Dental decay is a subject that should be constantly kept before the professional mind, and also, on account of its *humanitarian* aspect, in the professional *heart*. *To save* the natural teeth should be the mighty mission of dental science and art. Let the subject of decay of teeth and its treatment be agitated, and let it be presented to the consideration of all in every light of which it is capable of being presented. Every ray of light, every particle of truth upon this important and intricate subject, is earnestly craved by those among us who have "honest hearts and noble spirits" animating their bosoms. Even though we have no *new* ideas, no *startling* discoveries to herald forth, let us *counsel* together and animate each other to our utmost in perfecting ourselves for the proper discharge of duty in the treatment of the natural organs. Is the true *nature* of decay fully comprehended? Is it clearly understood? Or is dental *decay* yet an *enigma?* If we say, *oh yes*, decay is "*a chemical phenomenon, the causes of which are all*, and *always, apart from life*," does *this* strip the "protean disease" of all complexity? of all mystery? If we say, certainly we understand what *decay* is,—it is a "*chemico—vital phenomenon*," it is a *disease*, the *cause* or *causes* of which are constitutional and chemical, are we much better off? Have we divested the "fantastic disease" of its intrinsic obscurity? Have we removed the difficulties attending its study and its management? Ah, no.

Whether we be *doctors* "puffed up by our collegiate pride;" or whether *professors* "sitting on our scientific thrones;" whether we be *practitioners*, "humble and lowly," we are all put to our *wit's end* to comprehend all the phenomena we meet with in our observations of the "disease singularly called CARIES," and we all have the mortification at times to see that our most skillful efforts are abortive in their attempts to arrest and conquer the forces of the "Enemy!"

We are yet *pupils* seeking knowledge on this great subject. We deprecate "false doctrines." We would be free from false opinions. These often lead us to "injurious operations and to inefficacious treatments." We dislike vague words—hate loose language—loathe careless terms: these often generate and foster false doctrines and erroneous opinions. We are wedded to no "varieties." We *would be* wedded to no "absolute affirmations." We give up cherished *species*—be they "twenty," or be they the lingering "three," *black—brown*—"*and then white.*" We *would be* given up to no "*single cause—the action of one or several acids.*" *Whatever* "the destruction of a tooth is due to," that we want to know—*whatever* the salvation of a tooth is due to, that we want to do! Brethren, let us try to help each other to the attainment of these "*whatevers.*"

If decay has not its several varieties or its distinct forms, it has its several stages and its distinctive conditions. Each and every case of decay is a problem to be solved—a theorem to be demonstrated. The dentist must therefore be somewhat geometrical. He must understand his *propositions* before he can enunciate them or demonstrate them. He must be well versed in all *definitive* and *axiomatic* principles. He must be familiar with, and appreciative of, all *points*, *planes*, *angles*, *circles*, *cubes*, *squares*, *polygons*, *cylinders*, and *solids.* He must define clearly where "superficial" decays end, and where "deep seated decays" begin. He must study the *conditions* of the disintegrated tissues. He must settle the "quality" of the *integrate* tissues. He must define the metes and bounds of every thing comprehended in, and essential to, a perfect demonstration of the problem or theorem proposed. *Then he demonstrates!*

The primary or first stages of decay are *epienamel* and *subenamel* decays.

Enamel decays might be with some propriety distinguished as *true* and *false* enamel affections. For instance, a decay may have the outward appearance of being purely epienamel, but close inspection discloses the fact that the subjacent dentine is materially involved. This would be the case in all likelihood where the weakness and susceptibility of the dentine constitute a predisposing condition of that tissue. Again, the surface or even the body of the enamel may be materially affected, and yet the dentine and also the integrate portions of the enamel, be found firm and healthy. This condition of an enamel decay might be distinguished as *true* or *pure* enamel decay, whilst the former condition might be distinguished as *false* enamel decay.

Incipient *true* enamel decay is a mere *roughening* of the epienamel. If this incipient evil were not so much neglected, numerous cases would necessarily be presented for treatment, and they could be very effectually *nipped in the bud* by the simple operation of *filing*. Persons understanding true dental *economy* do not neglect these primary wants of the teeth. Prejudice against the *file*, and sheer ignorance cause many to neglect their teeth, until decay has well nigh consummated their destruction. This is a good thing for the "*tooth maker*," but it is death to the teeth. *Verbum sat sapientibus.*

Looking over our *case book* we count up *eighteen* teeth filed for *true epienamel* decay in the past *twenty* days, which is about the average of our ordinary experience in such decays.

We will enumerate them: Miss E. R., age 15; proximals of four upper incisors. Texture of enamel excellent—no question as to success. Miss B. S., age 13; all proximals of upper incisors, except those between the centrals which are plugged. Enamel in this case was pretty good. Decay obliterated entirely—consider the teeth safe. Miss M. S., age 18; one upper incisor filed and left sound. Miss W. R., age 22; one canine tooth above and two lateral incisors. Some teeth we plugged—found dentine only tolerably resistant and firm, yet strong hopes of success. Mrs. W., aged about 28; filed two lower bicuspids, and left the teeth in a very favorable condition. Mr. N. G., about 35; filed one upper second bicuspid for *infradentinal** decay, and the adjoining first molar for true epienamel decay. This gentleman has teeth of the finest possible texture. Miss M. N., age about 17; filed two superior incisors, the teeth being of a brittle structure, yet the case promises success. In this time we treated the incisors for Miss T., age 12, for *false* enamel decay. Also the incisors for Master N., age 15, for the same. In each case, the parties concerned thought "*cleaning*" was all the teeth needed. The *enamel* was apparently unaffected, but *subenamel* and *epidentinal* decay existed which we treated.

---

* We mention the filing of this *bicuspid* here, for *infradentinal* decay, to observe that Mr. G. objected to our filing his teeth. He told us he once had a lower jaw tooth filed and he could never afterwards eat on that tooth in consequence of the food's pressing on the gum, which became so annoying to him that he had the tooth extracted. He feared the same result if we should file these teeth. The condition of the bicuspid and the first molar was such, however, that plugging was out of the question. The teeth were in close proximity. The molar was slightly decayed far up on the palatine approximal angle, and the bicuspid was decayed from angle to angle on the post-proximal surface, leaving thin fringes of enamel around the broad cavity. The bottom of the cavity was hard and firmly consolidated dentine. We explained to Mr. G. the difference between a V-like cut and the cut we proposed to make, and assured him that his gum should be protected. He consented to our filing, and we accordingly filed the bicuspid so as to leave the buccal angles of the two teeth as near together as possible, the space widening towards the palatine angles. The grinding surface of the bicuspid was preserved as much as was possible, sufficiently so indeed to prevent particles of food of a size that could annoy from being compressed or rammed up against the gum.

